# MYC Enhances Cholesterol Biosynthesis and Supports Cell Proliferation Through SQLE

**DOI:** 10.3389/fcell.2021.655889

**Published:** 2021-03-11

**Authors:** Fan Yang, Junjie Kou, Zizhao Liu, Wei Li, Wenjing Du

**Affiliations:** State Key Laboratory of Medical Molecular Biology, Department of Cell Biology, Institute of Basic Medical Sciences Chinese Academy of Medical Sciences, School of Basic Medicine Peking Union Medical College, Beijing, China

**Keywords:** cholesterol synthesis, cell proliferation, cancer, SQLE, MYC

## Abstract

Oncogene c-*Myc* (referred in this report as *MYC*) promotes tumorigenesis in multiple human cancers. MYC regulates numerous cellular programs involved in cell growth and cell metabolism. Tumor cells exhibit obligatory dependence on cholesterol metabolism, which provides essential membrane components and metabolites to support cell growth. To date, how cholesterol biosynthesis is delicately regulated to promote tumorigenesis remains unclear. Here, we show that MYC enhances cholesterol biosynthesis and promotes cell proliferation. Through transcriptional upregulation of SQLE, a rate-limiting enzyme in cholesterol synthesis pathway, MYC increases cholesterol production and promotes tumor cell growth. SQLE overexpression restores the cellular cholesterol levels in *MYC-*knockdown cells. More importantly, in *SQLE*-depleted cells, enforced expression of MYC has no effect on cholesterol levels. Therefore, our findings reveal that SQLE is critical for MYC-mediated cholesterol synthesis, and further demonstrate that SQLE may be a potential therapeutic target in MYC-amplified cancers.

## Introduction

*MYC* is one of the most broadly deregulated oncogenes in human cancers (Dang, 2012). It is frequently translocated in multiple myelomas and is commonly found amplified among different human cancers (Shou et al., 2000; Zack et al., 2013; Annibali et al., 2014). MYC protein mediates its effects mainly through inappropriate regulation of transcriptional programs involved in a variety of biological processes, contributing to almost every aspect of tumorigenesis (Meyer and Penn, 2008). Indeed, *MYC* deletion inhibits cell growth such as T-cell leukemia (Sharma et al., 2006) and gastric cancer (Dong et al., 2019). MYC as a general transcription factor binds around 10–15% of all promoter regions (Li et al., 2003). Tumor cells require rapid biomass accumulation and high-fidelity DNA replication to sustain uncontrolled proliferation. MYC has been shown to activate metabolic genes involved in glucose and glutamine metabolism, as well as lipid and nucleotide biosynthesis, contributing to metabolic reprogramming (Ahuja et al., 2010; Morrish et al., 2010; Dang, 2013).

Cholesterol is vital for the survival and growth of tumor cells. It is produced via cholesterol biosynthesis pathway which involves two rate-limiting enzymes, 3-hydroxy-3-methylglutarylcoenzyme A reductase (HMGCR) and squalene monooxygenase (SQLE) (Luo et al., 2020). Cholesterol is an essential component of cell membrane to maintain its fluidity and impact intracellular signal transduction. In addition, cholesterol also serves as a precursor for steroid hormone, bile acids, and specific vitamins (Riscal et al., 2019). Due to its importance, intracellular cholesterol homeostasis is delicately controlled. Indeed, imbalanced cholesterol levels have strong associations with the risk of cardiovascular diseases (Luo et al., 2019; Wong et al., 2019). Cancer cells require high levels of cholesterol for membrane biogenesis and other functional needs (Huang et al., 2020). Based on TCGA database, increased activity of the cholesterol synthesis pathway is correlated with poor patient survival in sarcoma, acute myeloid leukemia, and melanoma (Kuzu et al., 2016). Besides, at least one gene expression in the cholesterol synthesis was increased among approximately 60% melanomas (Kuzu et al., 2016). Conversely, inhibition of cholesterol metabolism hinders tumor growth and invasion in a variety of cancers (Li et al., 2017; Costa et al., 2018).

SQLE is recognized as one of the rate-limiting enzymes in cholesterol biosynthesis pathway, which catalyzes squalene oxidization (Gill et al., 2011). It is reported that SQLE promotes tumor development (Cirmena et al., 2018; Liu et al., 2018; Xu et al., 2020). Several drugs against SQLE have been evaluated in anti-tumor trials (Cirmena et al., 2018; Liu et al., 2018). Recent study has reported that SQLE can be regulated at transcriptional, translational, and post-translational levels (Chua et al., 2020). Here we report that MYC binds to and activates SQLE promoter. Through transcriptional upregulation of SQLE, MYC increases cellular cholesterol levels and promotes cell proliferation. We also provide evidence that SQLE is critical for MYC-regulated cholesterol biosynthesis. Thus, our findings suggest that SQLE may be a potential therapeutic target in MYC-driven cancers.

## Materials and Methods

### Antibodies

Antibodies against β-Actin (#66009-1, dilution: 1/3000) and antibodies against SQLE (#12544-1-AP, dilution: 1/500) were purchased from Proteintech (United States). Antibodies against MYC (#ab32072, dilution: 1/1000) were purchased from Abcam (United States, dilution: 1/500).

### Cell Culture and Transfection

Human osteosarcoma cell line U2OS, human hepatocellular carcinoma cell line HepG2, human lung cancer cell line H1299, human colorectal cancer cell line SW480, human clear cell carcinoma cell line Caki-1, and human renal epithelial cell line HEK293T were obtained from the American Type Culture Collection (ATCC, United States). U2OS, HepG2, SW480, Caki-1, and HEK293T cell lines were maintained in Dulbecco’s modified Eagle’s medium (DMEM), and H1299 cell line was cultured in RPMI 1640 medium. All mediums were supplemented with 10% fetal bovine serum (FBS) plus 1% penicillin and streptomycin (P/S). All cells were cultured at 37°C in a humidified incubator with 5%CO_2_. All the growth mediums, FBS, and supplemental reagents were obtained from CELL technologies (United States).

The following siRNAs were purchased from GenPharma (China). siRNA sequences are listed below:

Control siRNA: 5′-UUCUCCGAACGUGUCACGUTT-3′;

*hMYC* siRNA#1: 5′-GCUCAUUUCUGAAGAGGACTT-3′;

*hMYC* siRNA#2: 5′-GGCGAACACACAACGUCUUTT-3′;

*hSQLE* siRNA#1: 5′-GCCUCUAAAUCUUUAGGUUTT-3′;

*hSQLE* siRNA#2: 5′-GCCCAGGUUGUAAAUGGUUTT-3′.

siRNAs were transfected into cells using Lipofectamine RNAiMAX transfection reagent (Invitrogen, United States) following the manufacturer’s instruction.

### Plasmids and Lentiviral Transduction

Two overexpression plasmids for MYC and SQLE were generated by cloning the ORF into the pRK5-Flag and pRK5-HA vectors separately. A lentiviral overexpression plasmid for MYC was generated by cloning the ORF into the pLV vector. Lentiviral particles were generated in HEK293T cells with cotransfection of the packaging vectors. Then, virus-containing supernatants were harvested and applied to infect the target cells with 8 μg/ml Polybrene (Santa Cruz Biotechnology, United States). Two days after the infection, the infected cells were cultured in the presence of 2 μg/ml puromycin (Sigma–Aldrich, United States) for 3 days. Finally, puromycin-resistant cells were pooled.

### Semi-Quantitative PCR With Reverse Transcription and Quantitative Real-Time PCR

Total RNA was isolated from cells by Trizol reagent (TIANGEN, China), and 1 μg RNA of each sample was reversed to cDNA by the First-Strand cDNA Synthesis System (TIANGEN, China). cDNA (0.025 μg) of each sample was used as a template to perform PCR. The primer pairs for human genes were:

MYC-F: 5′-GGCTCCTGGCAAAAGGTCA-3′

MYC-R: 5′-CTGCGTAGTTGTGCTGATGT-3′;

SQLE-F: 5′-TGACAATTCTCATCTGAGGTCCA-3′

SQLE-R: 5′-CAGGGATACCCTTTAGCAGTTTT-3′;

β-Actin-F: 5′-GACCTGACTGACTACCTCATGAAGAT-3′

β-Actin-R:5′-GTCACACTTCATGATGGAGTTGAAGGT-3′.

### Chromatin Immunoprecipitation and Reporter Assays

For chromatin immunoprecipitation assays, HEK293T cells were crosslinked with 1% formaldehyde for 15 min at room temperature and crosslinking was stopped by the addition of 125 nM glycine (final concentration). Cell lysates were sonicated for 3 cycles to generate DNA fragments with an average size between 150 and 900 bp, and immunoprecipitated with IgG and MYC antibodies. Bound DNA fragments were eluted and amplified by PCR. Primer pairs were:

RE1-F: 5′-GCTATGCCGCGTTTGGCCAATC-3′ (−1079/−1058)

RE1-R: 5′-CCTGAGCCCCGCCCCCGGTCCC-3′ (−950/−929);

RE2-F: 5′-GGATCACTTAAGGTCAGGAGTT-3′ (+1271/+1292)

RE2-R: 5′-GAATGGAAGCAGGGCTATCAGGC-3′ (+1414/+1436);

β-Actin-F: 5′-GGGTCTGCGCTGTAAGAGTT-3′

β-Actin-R: 5′-GAACTCAGCCAAGGGGACTC-3′.

For reporter assay, the *SQLE* genomic fragments (C6849–C7006) containing either the wild-type RE1 (5′-GGGCAGCACGCGGGGCG-3′) or mutant [(5′-GGGAAGCAAATCTGGGCG-3′), with mutated nucleotides underlined] and the *SQLE* genomic fragments (C9190–C9364) containing the wild-type RE2 (5′-CCACGGCGCCTGGCCTG-3′) binding region were cloned into pGL3-basic vector. Luciferase reporter assays were performed as described. Briefly, the reporter plasmids were transfected into HEK293T cells together with a Renilla luciferase plasmid and a plasmid expressing MYC protein. Twenty-four hours after transfection, the luciferase activity was determined using a Dual Luciferase Assay System (Promega, United States). Transfection efficiency was normalized on the basis of the Renilla luciferase activity.

### Cell Viability and Proliferation Assay

For cell viability assays, cells were transfected with siRNAs or plasmids for 48 h and seeded in 96-well cell culture dishes in triplicate at a density of 1000 cells per well. Two days later, CCK8 (Pplygen, China) was added into culture dishes for 3 h. OD_450 *nm*_ was measured to test cell viability by using microplate reader Flexstation 3 (Molecular Devices, United States). Relative OD_450 *nm*_ was calculated using the corrected sample reading.

Cell proliferation assays were performed as described. Briefly, cells were transfected with siRNAs or plasmids for48 h and seeded in six-well cell culture dishes in triplicate at a density of 40,000 cells per well. The medium was changed every day. Cell number at the indicated time points was counted using a Cell Counter (Countstar, China).

### Western Blotting

Whole-cell lysates were made in modified RIPA lysis buffer(10 mM Tris–HCl at pH 7.5, 5 mM EDTA, 150 mM NaCl, 1% NP-40, 1% sodium deoxycholate, 0.025% SDS, and 5 mM protease inhibitors) for 30 min on ice, and boiled in 2× loading buffer. Protein samples were resolved by SDS–PAGE and transferred onto PVDF membrane, which was blocked in 5% BSA and probed with the indicated antibodies.

### Statistical Analysis

Statistical significance was analyzed by two-tailed unpaired *t*-test and expressed as a *p*-value.

## Results

### Myc Enhances Intracellular Cholesterol Levels and Promotes Cell Proliferation

**FIGURE 1:**
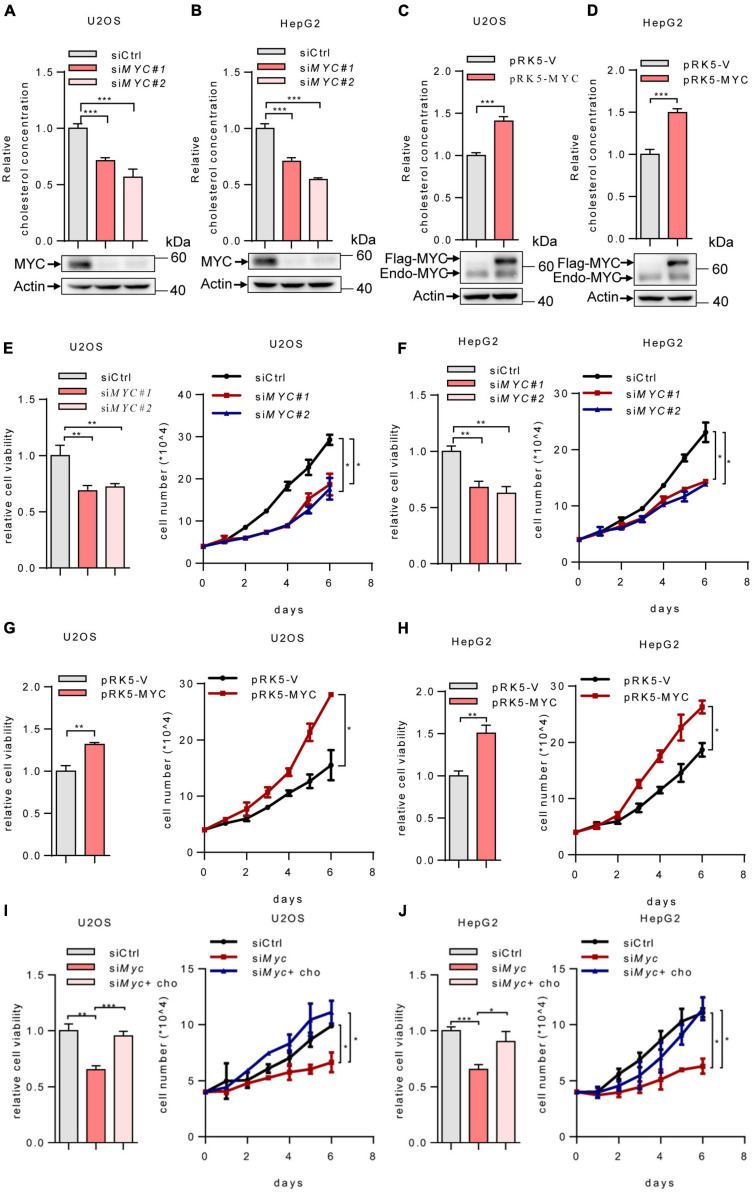
MYC increases intracellular cholesterol levels and promotes cell proliferation. **(A,B)** U2OS and HepG2 cells were transfected with control or two different sets of *MYC* siRNAs. Cells were cultured in the lipoprotein-depleted fetal bovine serum (LPDS) medium. Intracellular cholesterol levels (top) and corresponding protein expression (bottom) were shown. **(C,D)** U2OS cells and HepG2 cells cultured in LPDS medium were transfected with plasmid expressing the MYC protein. Intracellular cholesterol levels (top) and corresponding protein expression (bottom) were shown. **(E,F)** Relative cell viability (left) and cell proliferation (right) in control and *MYC* knockdown U2OS cells and HepG2 cells. Relative cell viability was measured with CCK8. **(G,H)** Relative cell viability (left) and cell proliferation (right) in control and MYC overexpressing U2OS cells and HepG2 cells. Relative cell viability was measured with CCK8. **(I,J)** Relative cell viability (left) and cell proliferation (right) of U2OS and HepG2 *MYC*-knockdown cells cultured in LPDS medium with or without 20 μg/ml cholesterol. Relative cell viability was measured with CCK8. In **(A–H)**, *n* = 3 independent experiments. Data are means ± SD. Statistical significance was determined by two-tailed unpaired *t*-test. *P < 0.05, **P < 0.01, and ***P < 0.001.

Previous studies have revealed that MYC is required for the upregulation of mevalonate pathway in certain cancer cells (Wang et al., 2017). To further investigate the role of MYC in cholesterol biosynthesis, we knocked down *MYC* in human osteosarcoma U2OS cells and human hepatoma HepG2 cells. *MYC* depletion decreased intracellular cholesterol levels (Figures 1A,B). Conversely, MYC overexpression in U2OS and HepG2 cells led to a noticeable increase in cholesterol levels (Figures 1C,D). These results indicate that MYC enhances intracellular cholesterol levels. Furthermore, silencing of *MYC* inhibited cell viability and cell proliferation (Figures 1E,F), whereas overexpression of MYC promoted cell viability and proliferation in both U2OS and HepG2 cells (Figures 1G,H). We next knocked down MYC in U2OS and HepG2 cells and cultured cells in lipid-free medium in the presence or absence of cholesterol (Figures 1I,J). Cholesterol addition rescued the decreased cell viability and cell proliferation caused by MYC knockdown. These data indicated that MYC supports tumor cell growth through cholesterol. Taken together, these data indicate that MYC promotes cholesterol synthesis and cell proliferation.

### MYC Transcriptionally Upregulates SQLE Expression

**FIGURE 2:**
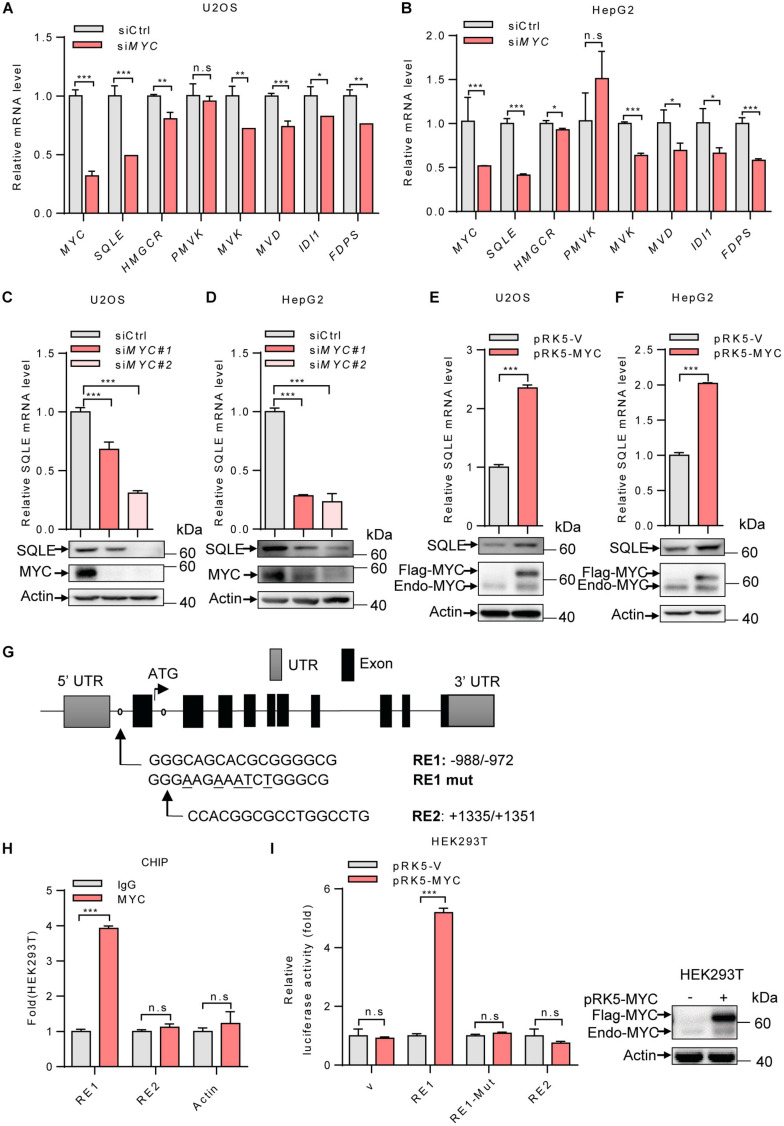
MYC transcriptionally upregulates the expression of SQLE. **(A,B)** U2OS cells and HepG2 cells were transfected with control or *MYC* siRNA. The mRNA levels of seven metabolic enzymes in cholesterol synthesis pathway were detected by qPCR. **(C,D)** SQLE mRNA (top) and protein (bottom) expression in control and *MYC* knockdown U2OS and HepG2 cells. **(E,F)** *SQLE* mRNA (top) and protein (bottom) expression in U2OS and HepG2 cells expressing ectopic MYC or control protein. **(G)** Schematic representation of human SQLE genomic structure. Shown are the putative MYC response elements (RE1/RE2), and the mutant response element (RE1-mut, with mutated nucleotides underlined). Arrows mark the positions of the primers used for qPCR in the ChIP assay. UTR, untranslated region. **(H)** HEK293T cells were analyzed by ChIP assay using an anti-MYC antibody or rabbit IgG control. **(I)** Luciferase assays performed using reporter constructs containing the wild-type or mutant MYC response element were transfected into HEK293T cells, together with or without ectopically expressed MYC. Renilla vector was used as a transfection internal control. Relative levels of luciferase are shown (left). MYC expression in HEK293T cells was detected (right). In **(A–F,H,I)** *n* = 3 independent experiments. Data are means ± SD. Statistical significance was determined by two-tailed unpaired *t*-test. *P < 0.05, **P < 0.01, and ***P < 0.001.

To investigate the mechanism by which MYC regulates the cholesterol biosynthesis, we analyzed the mRNA levels of several enzymes involved in cholesterol metabolism. *MYC-*knockdown decreased expression of genes in cholesterol synthesis, while SQLE expression was mostly reduced in *MYC*-depleted cells (Figures 2A,B). SQLE is a rate-limiting enzyme in cholesterol biosynthesis. We next examined how MYC regulates SQLE expression. We knocked down expression of MYC using two different sets of siRNAs in both U2OS and HepG2 cells. As shown in Figures 2C,D, both protein and mRNA levels of SQLE decreased in *MYC* knocking down cells compared to control cells (Figures 2C,D). Similar results were observed in the other three cell lines including H1299, SW480, and Caki-1 cells (Supplementary Figures S1A–C). Conversely, enforced expression of MYC promoted SQLE expression in both mRNA and protein levels (Figures 2E,F and Supplementary Figures S1D–F). These data suggest that MYC upregulates SQLE expression.

Next, to determine whether SQLE is a transcriptional target of MYC, we analyzed the human *SQLE* gene sequence for potential MYC protein response elements in JASPAR database. We identified two putative MYC response elements (RE1 and RE2) (Figure 2G). To investigate the binding of MYC to these two response elements, we performed ChIP-quantitative PCR (ChIP-qPCR) experiments. As shown in Figure 2H, MYC bound to the genomic region RE1 of *SQLE* gene, but not RE2 (Figure 2H). To evaluate whether the response element within *SQLE* confers MYC-dependent transcriptional activation, we cloned DNA fragments containing the wild-type response element (RE1) or a mutant response element (RE1-mut) into luciferase reporter plasmid. MYC was able to induce luciferase expression from the RE1 plasmid, but not from the RE1-mut plasmid (Figure 2I). These data suggest that SQLE is a transcriptional target of MYC and MYC activates SQLE expression in a transcriptional manner.

### MYC Regulates Cellular Cholesterol Levels Through SQLE

**FIGURE 3:**
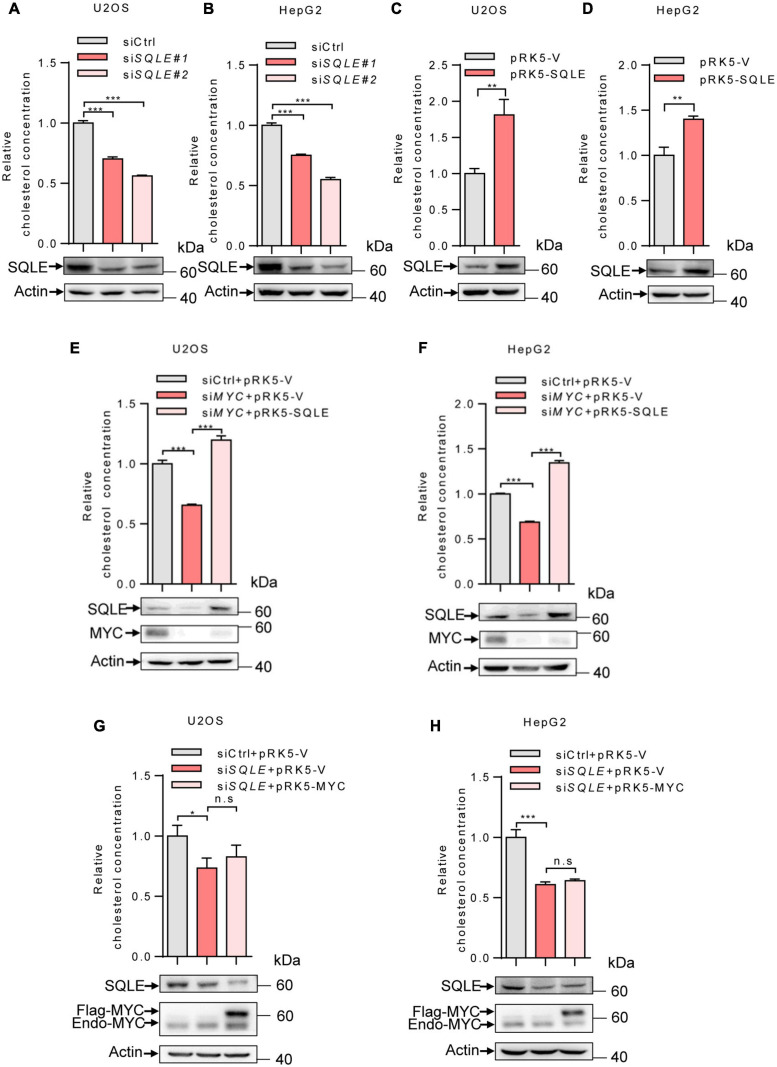
MYC increases intracellular cholesterol levels through SQLE. **(A,B)** U2OS and HepG2 cells were transfected with control or different sets of *SQLE* siRNAs. Cells were cultured in the LPDS medium. Intracellular cholesterol levels (top) and corresponding protein expression (bottom) were detected. **(C,D)** U2OS cells and HepG2 cells cultured in LPDS medium were ectopically expressed the SQLE protein. Intracellular cholesterol levels (top) and corresponding protein expression (bottom) were shown. **(E,F)** Intracellular cholesterol levels (top) and protein expression (bottom) in U2OS and HepG2 cells transfected with control or *MYC* siRNA, with or without Flag-SQLE as indicated. **(G,H)** U2OS cells and HepG2 cells were transfected with control or *SQLE* siRNA, and then enforced expression of MYC into *SQLE* knockdown cells. Intracellular cholesterol levels (top) and protein expression (bottom) were examined, respectively. For cholesterol content detection, cells were cultured in the medium with LPDS. In **(A–H)** 2 μg plasmids and 1 μg siRNAs were used in all experiments. *n* = 3 independent experiments. Data are means ± SD. Western blots represent three independent experiments. Statistical significance was determined by two-tailed unpaired *t*-test. *P < 0.05, **P < 0.01, and ***P < 0.001.

We next determine the role of SQLE in MYC-mediated cholesterol synthesis. Inhibition of SQLE expression using two sets of siRNAs led to a decreased cholesterol level in both U2OS and HepG2 cells (Figures 3A,B). Conversely, SQLE overexpression increased cholesterol levels (Figures 3C,D). Furthermore, we enforced expression of SQLE in *MYC*-knocking down cells. *MYC* depletion reduced cellular cholesterol levels, while SQLE overexpression reversed it in both U2OS and HepG2 cells (Figures 3E,F). Surprisingly, MYC overexpression failed to restore the cholesterol levels in *SQLE* deficient cells (Figures 3G,H). Together, these results suggest that MYC stimulates cholesterol biosynthesis through SQLE.

### MYC Promotes Tumor Cell Growth Through SQLE

**FIGURE 4:**
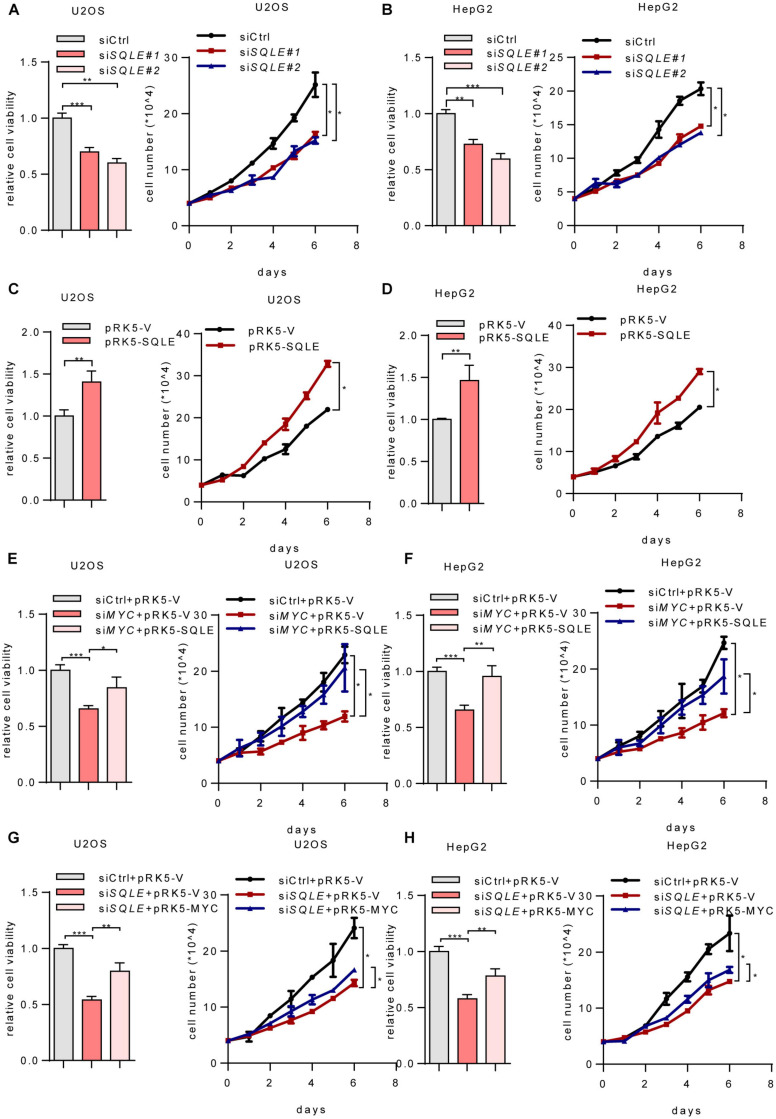
MYC activates SQLE expression to support tumor growth. **(A,B)** Relative cell viability (left) and cell proliferation (right) in control and *SQLE* knockdown U2OS cells and HepG2 cells. Relative cell viability was measured with CCK8. **(C,D)** Relative cell viability (left) and cell proliferation (right) in control and SQLE overexpressing U2OS cells and HepG2 cells. Relative cell viability was measured with CCK8. **(E,F)** U2OS and HepG2 cells were transfected with control or *MYC* siRNA in the presence or absence of exogenous SQLE. Cell viability (left) and cell proliferation (right) were detected, respectively. Relative cell viability was measured with CCK8. **(G,H)** U2OS and HepG2 cells were transfected with control or *SQLE* siRNA in the presence or absence of exogenous MYC. Cell viability (left) and cell proliferation (right) were detected, respectively. Relative cell viability was measured with CCK8. In **(A–H)**, 2 μg plasmids and 1 μg siRNAs were used in all experiments. *n* = 3 independent experiments. Data are means ± SD. Statistical significance was determined by two-tailed unpaired *t*-test. *P < 0.05, **P < 0.01, and ***P < 0.001.

Cholesterol metabolism provides essential membrane components as well as metabolites with a variety of biological functions (Huang et al., 2020). To verify whether SQLE is involved in MYC-mediated cell growth, we depleted *SQLE* using two sets of siRNAs. Depletion of *SQLE* repressed cell proliferation and cell viability (Figures 4A,B). Conversely, ectopically expressing SQLE promoted cell growth (Figures 4C,D). More importantly, ectopically expressing SQLE almost completely rescued the decreased cell viability and cell proliferation caused by *MYC* knockdown (Figures 4E,F). On the contrary, overexpression of MYC partially increased cell viability and proliferation in *SQLE*-knockdown cells (Figures 4G,H). These data indicate that MYC promotes cell growth at least partially through SQLE. Then we analyzed TCGA cohort to identify the correlation between MYC and SQLE expression (Supplementary Figure 2). MYC was positively correlated with SQLE in several cancers such as cervical squamous cell carcinoma and endocervical adenocarcinoma (CESC), colon adenocarcinoma (COAD), head and neck squamous cell carcinoma (HNSC), and Sarcoma (SARC). Collectively, these data indicate that SQLE is critical for MYC-mediated tumor cell proliferation.

## Discussion

MYC promotes metabolic reprogramming in cancer and is involved in lipogenesis by pronouncedly activating acetyl-CoA carboxylase (ACACA), fatty acid synthetase (FASN), and stearoyl-CoA desaturase (SCD) (Zeller et al., 2003; Loven et al., 2012). In patient-derived brain-tumor-initiating cells, MYC is required for upregulation of the mevalonate pathway. And this upregulation further elevates miR-33b to increase MYC expression and thus establishes a positive feedback loop (Wang et al., 2017). In this study, we report that MYC stimulates cholesterol production and promotes tumor cell proliferation via transcriptionally upregulating SQLE.

SQLE is one of the rate-limiting enzymes in cholesterol biosynthesis, and is one of the most significantly upregulated genes in numerous tumors (Xu et al., 2020). A pan-cancer genomic and transcriptomic analysis also highlights SQLE as a key player in maintaining cell survival under hypoxia (Bai et al., 1992). Additionally, in hepatocellular carcinoma (Liu et al., 2018) and breast cancer (Cirmena et al., 2018), SQLE expression is correlated with cancer stage and identified as a prognostic marker. Here we explore the possible correlation between MYC and SQLE. Interestingly, enforced expression of SQLE in *MYC*-depleted cells completely rescues intracellular cholesterol contents, while overexpression of MYC fails to affect the cholesterol levels in *SQLE*-deficient cells. This means that SQLE is indispensable for MYC-regulated cholesterol levels. Of note, we cannot rule out the possibility that MYC may also simultaneously activate the upstream enzymes in the mevalonate pathway.

Recent studies show that SQLE and MYC are co-localized to the same amplicons (Brown et al., 2016; Wolpaw and Dang, 2018). And amplification of SQLE is thought to be associated with amplification of the oncogene MYC (Parris et al., 2014). Our work finds that MYC transcriptionally upregulates SQLE expression. Although a study shows that SQLE expression is independent of MYC in breast and ovarian cancers (Brown et al., 2016), indicating cell heterogeneity in different contexts.

Cholesterol metabolism is essential for tumor growth. Normal mammalian cells meet their need for cholesterol through uptake or *de novo* synthesis (Goldstein and Brown, 1990). Many cancer cell lines depend on exogenous cholesterol for their growth. For example, when incubated in lipoprotein-depleted serum, U-937 cells die after 4 days unless supplemented with cholesterol (Garcia-Bermudez et al., 2019). ALK^+^ anaplastic large cell lymphoma (ALCL) cell lines and primary tumors which are less of SQLE expression are highly dependent on cholesterol uptake (Garcia-Bermudez et al., 2019). However, under lipid-free culture conditions, many cells still can proliferate suggesting that these cells may obtain sufficient cholesterol through *de novo* synthesis (Garcia-Bermudez et al., 2019). In addition, lower levels of LDLR but higher levels of SQLE are expressed in advanced-stage prostate cancer, revealing a greater dependence on cholesterol synthesis than uptake (Freudiger et al., 2008). As above, many tumor cells rely on cholesterol biosynthesis to support their growth. Because of its ubiquitous role in human tumors, MYC is an attractive therapeutic target. Cells with deregulated MYC expression often lose negative control and thus depend on a continual supply of nutrients, causing them to be nutrient addicted (Dang, 2012). Therefore, therapies targeting SQLE may be a promising strategy in the treatment of certain MYC-driven cancer.

## Data Availability Statement

The original contributions presented in the study are included in the article/Supplementary Material. Further inquiries can be directed to the corresponding author/s.

## Author Contributions

WD and FY designed the experiments and interpreted results. FY performed all experiments. ZL and WL provided technical assistance. WD supervised the research. WD and JK wrote the manuscript with the help of FY. All authors discussed the results and commented on the manuscript.

## Conflict of Interest

The authors declare that the research was conducted in the absence of any commercial or financial relationships that could be construed as a potential conflict of interest.

## Supplementary Material

Supplementary Figure 1 MYC promotes the expression of SQLE. **(A–C)** SQLE mRNA (Top) and protein (Bottom) expression in control and *MYC* knockdown H1299 cells, SW480 cells, and Caki-1 cells. **(D–F)** SQLE mRNA (Top) and protein (Bottom) expression in H1299 cells, SW480 cells, and Caki-1 cells expressing ectopic MYC or control protein. In **(A–F)**, 2 μg plasmids and 1 μg siRNAs were used in all experiments. *n* = 3 independent experiments. Data are means ± SD. Statistical significance was determined by two-tailed unpaired *t*-test. ^∗^*P* < 0.05, ^∗∗^*P* < 0.01, and ^∗∗∗^*P* < 0.001.

Supplementary Figure 2 TCGA cohort identified a correlation between MYC and SQLE expression. **(A–D)** The correlation between MYC and SQLE expression in cervical squamous cell carcinoma and endocervical adenocarcinoma (CESC), colon adenocarcinoma (COAD), head and neck squamous cell carcinoma (HNSC), and Sarcoma (SARC) by analyzing TCGA cohort.
