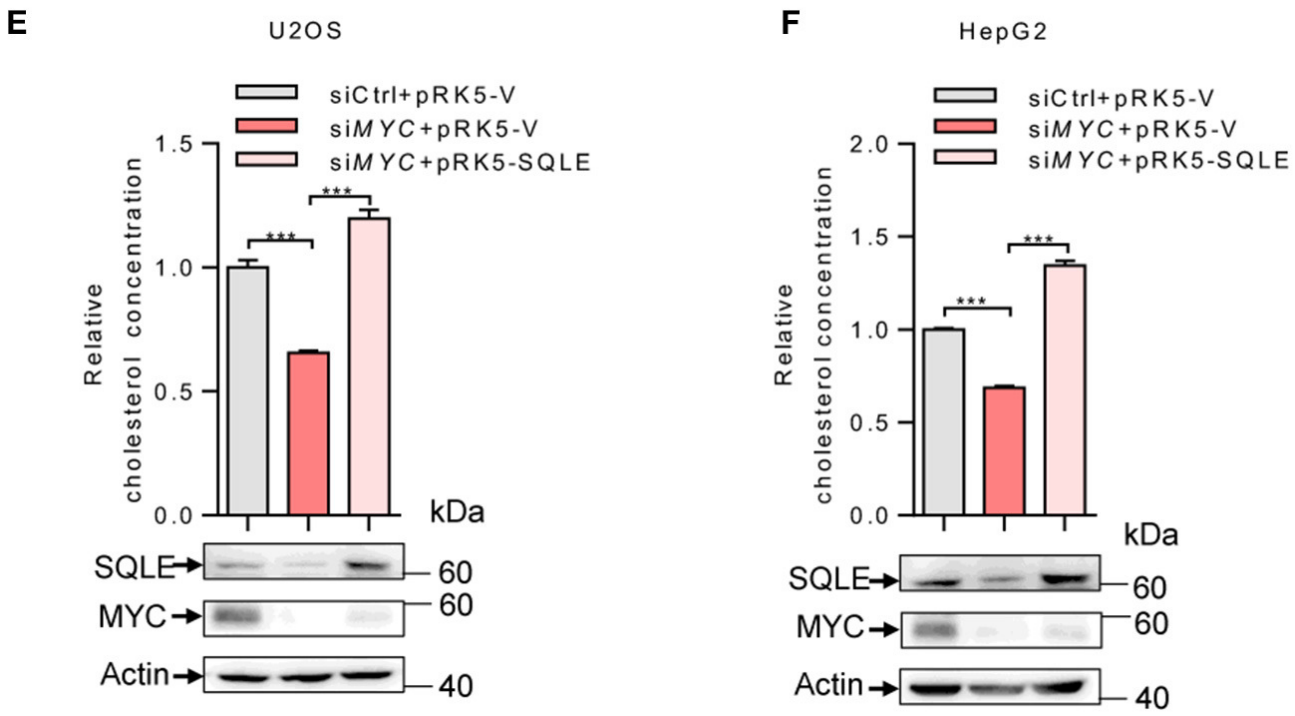# Corrigendum: MYC Enhances Cholesterol Biosynthesis and Supports Cell Proliferation Through SQLE

**DOI:** 10.3389/fcell.2021.705769

**Published:** 2021-06-09

**Authors:** Fan Yang, Junjie Kou, Zizhao Liu, Wei Li, Wenjing Du

**Affiliations:** State Key Laboratory of Medical Molecular Biology, Department of Cell Biology, Institute of Basic Medical Sciences Chinese Academy of Medical Sciences, School of Basic Medicine Peking Union Medical College, Beijing, China

**Keywords:** cholesterol synthesis, cell proliferation, cancer, SQLE, MYC

In the original article, there was a mistake in Figure 1I and Figure 1J as published. The statistical significance was calculated between the siCtrl and siMYC+cho group. However, the correct comparison is between siMYC and siMYC+cho group. The corrected Figure 1I and Figure 1J appear below.

**Figure 1:**
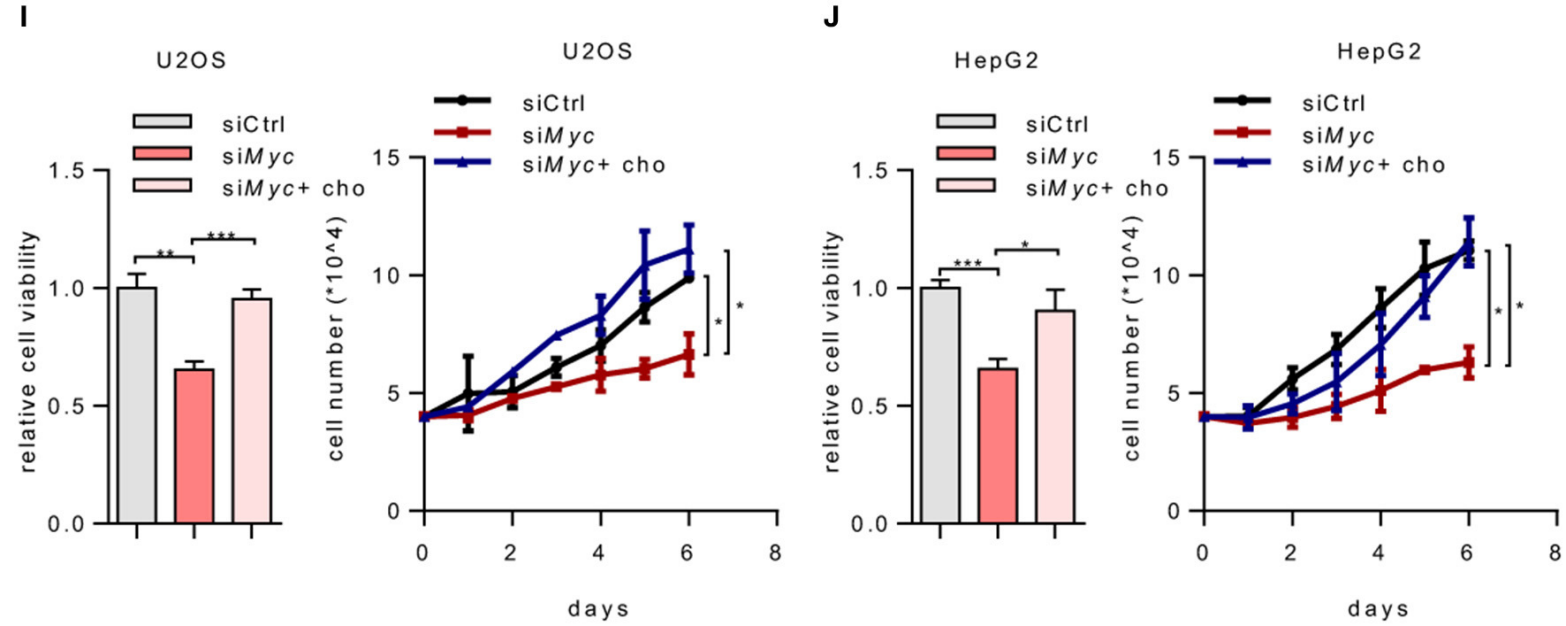


In the original article, there was a mistake in Figure 3E and Figure 3F as published. The statistical significance was calculated between the siCtrl+pRK5-V and siMYC+pRK5-SQLE group. However, the correct comparison is between siMYC+ pRK5-V and siMYC+pRK5-SQLE group. The corrected Figure 3E and Figure 3F appear below.

The authors apologize for these errors and state that this does not change the scientific conclusions of the article in any way. The original article has been updated.

**Figure 3:**