# Supplementary figures and images for: MYC Enhances Cholesterol Biosynthesis and Supports Cell Proliferation Through SQLE
Source: Front Cell Dev Biol. 2021 Mar 11;9:655889. doi: 10.3389/fcell.2021.655889 (PMC8006431; doi:10.3389/fcell.2021.655889)

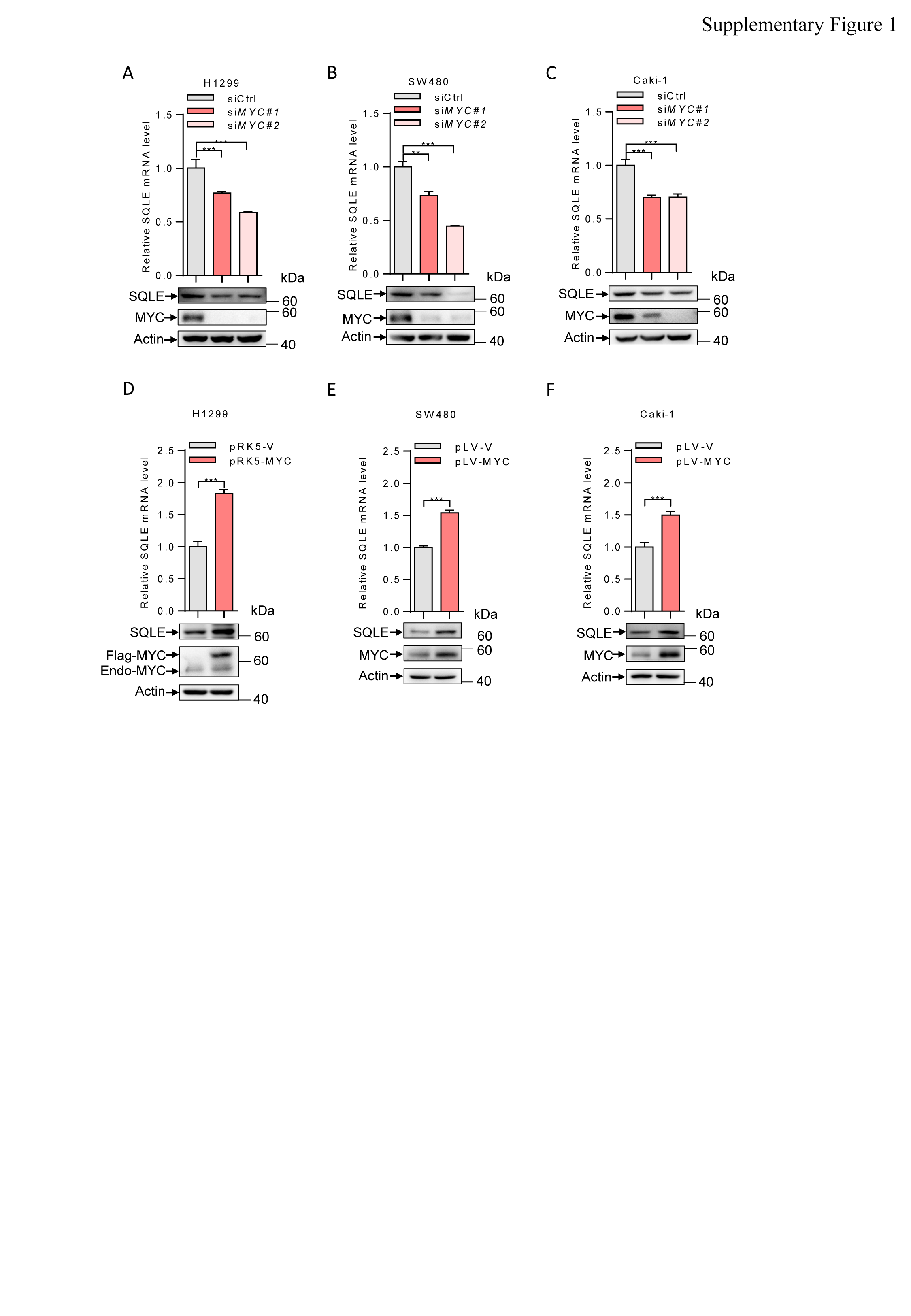

Supplement: Supplementary Figure 1 — MYC promotes the expression of SQLE. (A–C) SQLE mRNA (Top) and protein (Bottom) expression in control and MYC knockdown H1299 cells, SW480 cells, and Caki-1 cells. (D–F) SQLE mRNA (Top) and protein (Bottom) expression in H1299 cells, SW480 cells, and Caki-1 cells expressing ectopic MYC or control protein. In (A–F), 2 μg plasmids and 1 μg siRNAs were used in all experiments. n = 3 independent experiments. Data are means ± SD. Statistical significance was determined by two-tailed unpaired t-test. ∗P < 0.05, ∗∗P < 0.01, and ∗∗∗P < 0.001. [file Image_1.TIF]

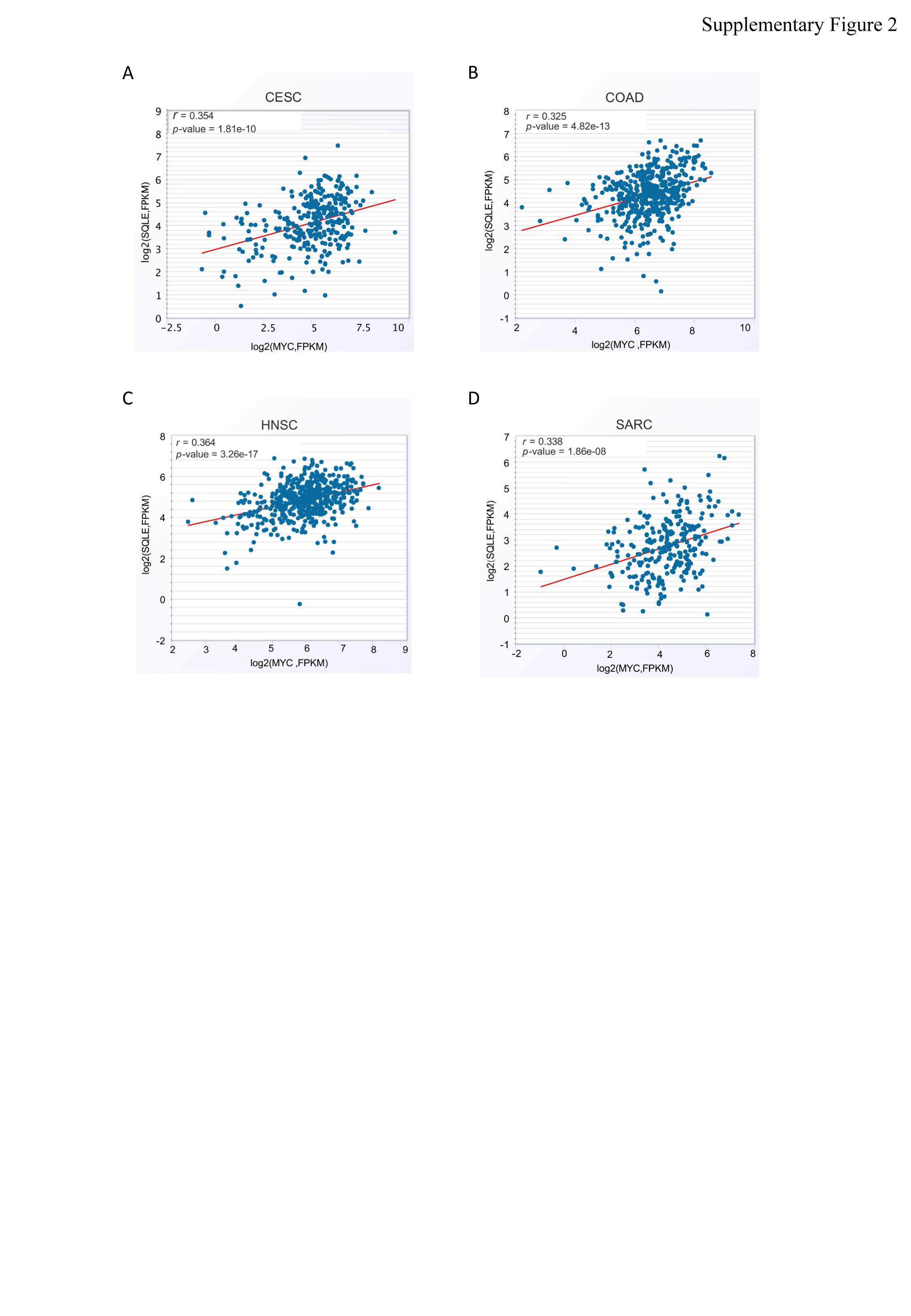

Supplement: Supplementary Figure 2 — TCGA cohort identified a correlation between MYC and SQLE expression. (A–D) The correlation between MYC and SQLE expression in cervical squamous cell carcinoma and endocervical adenocarcinoma (CESC), colon adenocarcinoma (COAD), head and neck squamous cell carcinoma (HNSC), and Sarcoma (SARC) by analyzing TCGA cohort. [file Image_2.TIF]
